# Progenitor cell-derived exosomes endowed with VEGF plasmids enhance osteogenic induction and vascular remodeling in large segmental bone defects

**DOI:** 10.7150/thno.50741

**Published:** 2021-01-01

**Authors:** Yao Zha, Yawu Li, Tianyi Lin, Jia Chen, Shengmin Zhang, Jianglin Wang

**Affiliations:** 1 Advanced Biomaterials and Tissue Engineering Center,Huazhong University of Science and Technology, Wuhan, 430074, P. R. China.; 2 Department of Biomedical Engineering, College of Life Science and Technology, Huazhong University of Science and Technology, Wuhan, 430074, P. R. China.

**Keywords:** engineered exosome, VEGF gene, anchor peptide, 3D printing, vascularized osteogenesis

## Abstract

Large segmental bone regeneration remains a great challenge due to the lack of vascularization in newly formed bone. Conventional strategies primarily combine bone scaffolds with seed cells and growth factors to modulate osteogenesis and angiogenesis. Nevertheless, cell-based therapies have some intrinsic issues regarding immunogenicity, tumorigenesis, bioactivity and off-the-shelf transplantation. Exosomes are nano-sized (50-200 nm) extracellular vesicles with a complex composition of proteins, nucleic acids and lipids, which are attractive as therapeutic nanoparticles for disease treatment. Exosomes also have huge potential as desirable drug/gene delivery vectors in the field of regenerative medicine due to their excellent biocompatibility and efficient cellular internalization.

**Methods:** We developed a cell-free tissue engineering system using functional exosomes in place of seed cells. Gene-activated engineered exosomes were constructed by using ATDC5-derived exosomes to encapsulate the VEGF gene. The specific exosomal anchor peptide CP05 acted as a flexible linker and effectively combined the engineered exosome nanoparticles with 3D-printed porous bone scaffolds.

**Results:** Our findings demonstrated that engineered exosomes play dual roles as an osteogenic matrix to induce the osteogenic differentiation of mesenchymal stem cells and as a gene vector to controllably release the VEGF gene to remodel the vascular system. *In vivo* evaluation further verified that the engineered exosome-mediated bone scaffolds could effectively induce the bulk of vascularized bone regeneration.

**Conclusion:** In our current work, we designed specifically engineered exosomes based on the requirements of vascularized bone repair in segmental bone defects. This work simultaneously illuminates the potential of functional exosomes in acellular tissue engineering.

## Introduction

Conventional tissue engineering consisting of biomaterial scaffolds and seed cells, and growth factors plays a pivotal role in inducing the regenerative repair of injured tissues and organs 1-4. Among the three basic elements of tissue engineering, the seed cells are absolutely vital for initiating tissue regeneration. However, cell-based tissue engineering simultaneously has a number of drawbacks related to the cell source and activity, immunological rejection, long therapeutic times and high costs in clinical application 5, 6. Thus, cell-free tissue engineering has been extensively explored in the field of regenerative medicine as a safe, effective and off-the-shelf strategy.

Cell-based therapies are advanced therapeutic strategies that have brought promise for some severe diseases 7-9. Unfortunately, cell-based therapy has still not become popularized in clinical applications. The safety and effectiveness of cell transplantation remains as issue of major focus. Exosomes derived from therapeutic cells contain many functional microRNAs, proteins and bioactive molecules that have biological effects in modulating cell behaviours and activating signalling pathways, as well as directly participating in the treatment of diseases 10-12. More importantly, the exosome itself is not a real cell and it can readily bypass the routine drawbacks of cell-based therapy 13-16. Consequently, exosome-mediated therapy has been considered to be an alternative to conventional cell therapy and has been used in cell-free tissue engineering.

Exosome-mediated acellular bone regeneration has been documented to enhance skull regeneration in our previously published work 17. Herein, we continue to investigate the potential of exosome-enhanced therapy on large segmental bone defects that cannot be repaired without the promotion of bone cells and the reconstruction of internal vasculature. To address the aforementioned requirements, a specifically engineered exosome has been constructed using ATDC5-derived exosomes to encapsulate a plasmid carrying the vascular endothelial growth factor (VEGF) gene. ATDC5 is a chondrogenic progenitor cell line that has been verified to exhibit significant osteogenic differentiation capacity 18. VEGF is a crucial growth factor that has been shown to remodel the vasculature in many regeneration tissues 19, 20. Hence, the well-designed, engineered exosomes exhibit dual roles as an osteogenic matrix and a gene vector to potentially increase vascularized osteogenesis in segmental bone defects.

**Figure 1:**
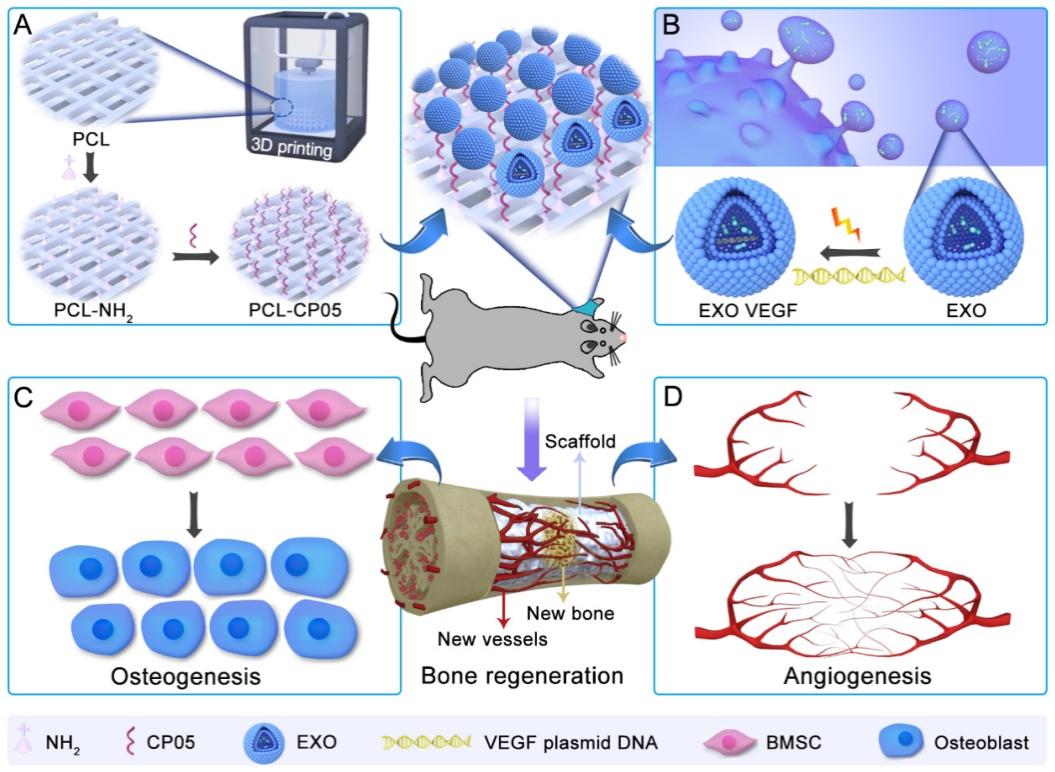
** General idea of engineered exosome enhanced therapies on osteogenesis and angiogenesis. (A)** 3D-printed porous PCL scaffolds were modified with 1,6-hexanediamine to generate the amino group on PCL scaffolds that were subsequently modified with the exosomal anchor peptide CP05. **(B)** Engineered exosomes were fabricated by encapsulating the VEGF plasmid DNA into ATDC5-derived exosomes. The well-designed bone scaffolds were constructed by combining the engineered exosomes with the CP05 modified 3D-printed scaffolds, and eventually implanted into a rat radial defect model to promote osteogenesis **(C)** and angiogenesis **(D)**.

Exosome-based therapy has been primarily performed via intravenous administration of exosomes designed with targeting molecules or the homing effects of stem cells 21-23. However, the intravenous administration of functional exosomes results in minimal accumulation at the defect site, and can also affect the obstruction risk of some blood-rich organs 24-26. Thus, in this work, we combined engineered exosomes with polycaprolactone (PCL) 3D-printed porous bone scaffolds to ensure sustainable and stable therapy at the local defect site. PCL, as an FDA-approved, biodegradable material, has been extensively applied in bone tissue engineering with minimal provocation of inflammatory and immunological responses, perfect biocompatibility and nontoxic degradation 27, 28. Noticeably, a flexible and specific connection between the engineered exosomes and the 3D-printed scaffolds is an important precondition required to realize effective topical therapies. The exosomal anchor peptide CP05 has been reported to specifically bind to the antigen CD63, which is a tetraspanin enriched on the exosome surface and has been used as an exosomal marker 29, 30. Hence, we used CP05 as a flexible linker to modify the 3D-printed scaffolds to promote the grafting efficiency of the engineered exosomes. The resulting exosome-activated bone scaffolds had dual functions in inducing osteogenic differentiation and remodelling vasculature formation *in vivo*** (Figure 1)**.

## Results

### Identification and characterization of exosomes

**Figure 2:**
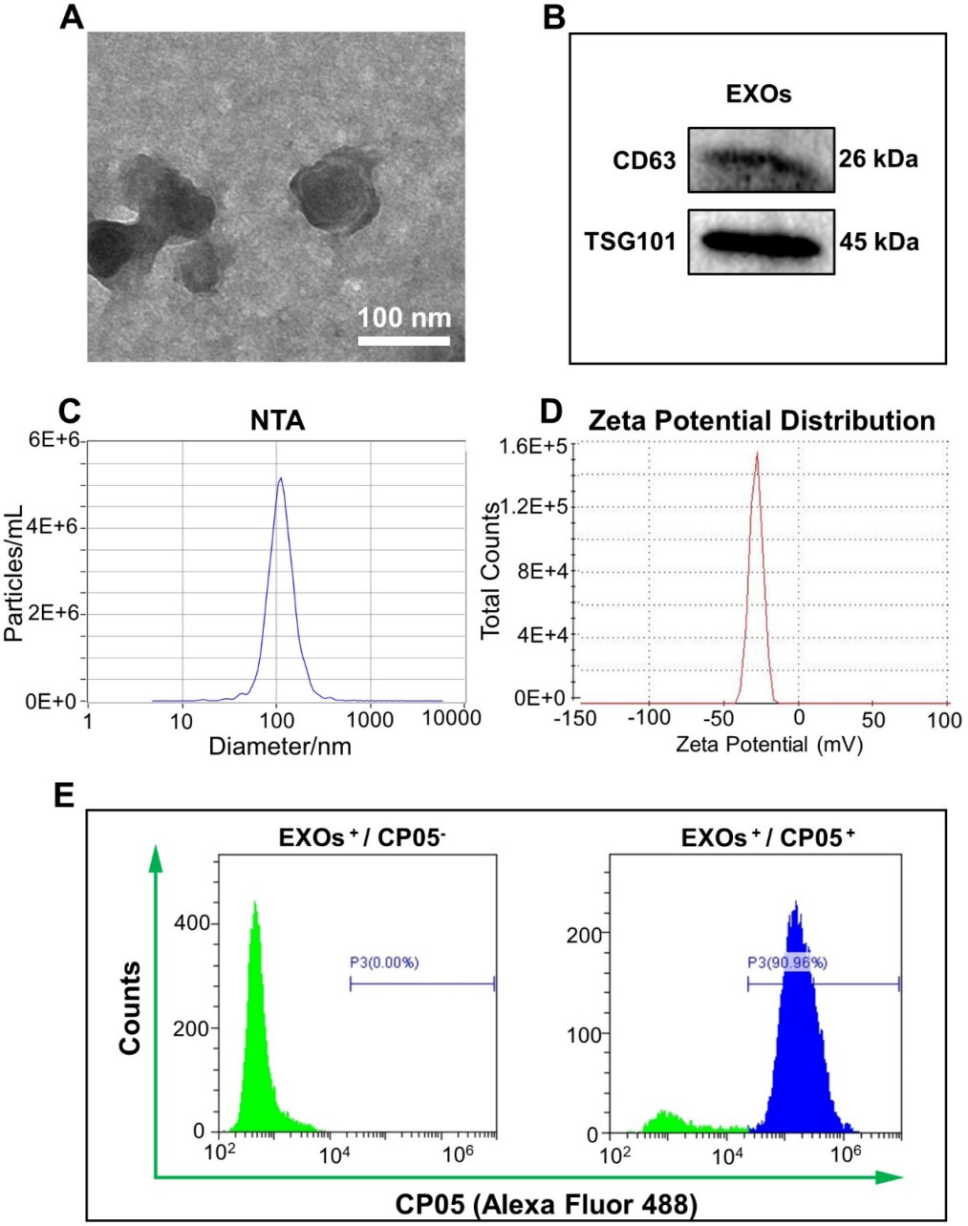
** Characterization of ATDC5-derived exosomes. (A)** TEM images of ATDC5-derived exosomes (EXOs) presented a spherical morphology with bi-layered membrane structure. **(B)** Two EXOs protein markers CD63 and TSG101 were verified by western blot. **(C)** NTA analysis showed that the size of nanoscale EXOs was about 114.2 nm (n = 3). **(D)** Zeta potential distribution of EXOs was about -32.0 mV by DLS (n = 3). **(E)** The grafting efficiency of CP05 to EXOs was as high as 90.96% based on the analysis of flow cytometry.

Based on observations of TEM images, exosomes derived from ATDC5 cells had a spherical morphology with a diameter of 100 nm **(Figure 2A)**. Two specific markers of exosome-related proteins, CD63 and TSG101, were detected by western blot **(Figure 2B)**. The size and size distribution of the exosomes was 114.2 ± 1.8 nm (n = 3), based on nanoparticle tracking analysis (NTA) measurements **(Figure 2C)**. The zeta potential of the exosomes was -32.0 ± 1.5 mV, based on the dynamic light scattering (DLS) analysis **(Figure 2D)**. Successful grafting of the CP05 exosomal anchor peptide with the exosomes was verified via flow cytometry, and the grafting efficiency was as high as 90.96% **(Figure 2E)**.

### Local expression of gene-activated engineered exosomes *in vitro*

**Figure 3:**
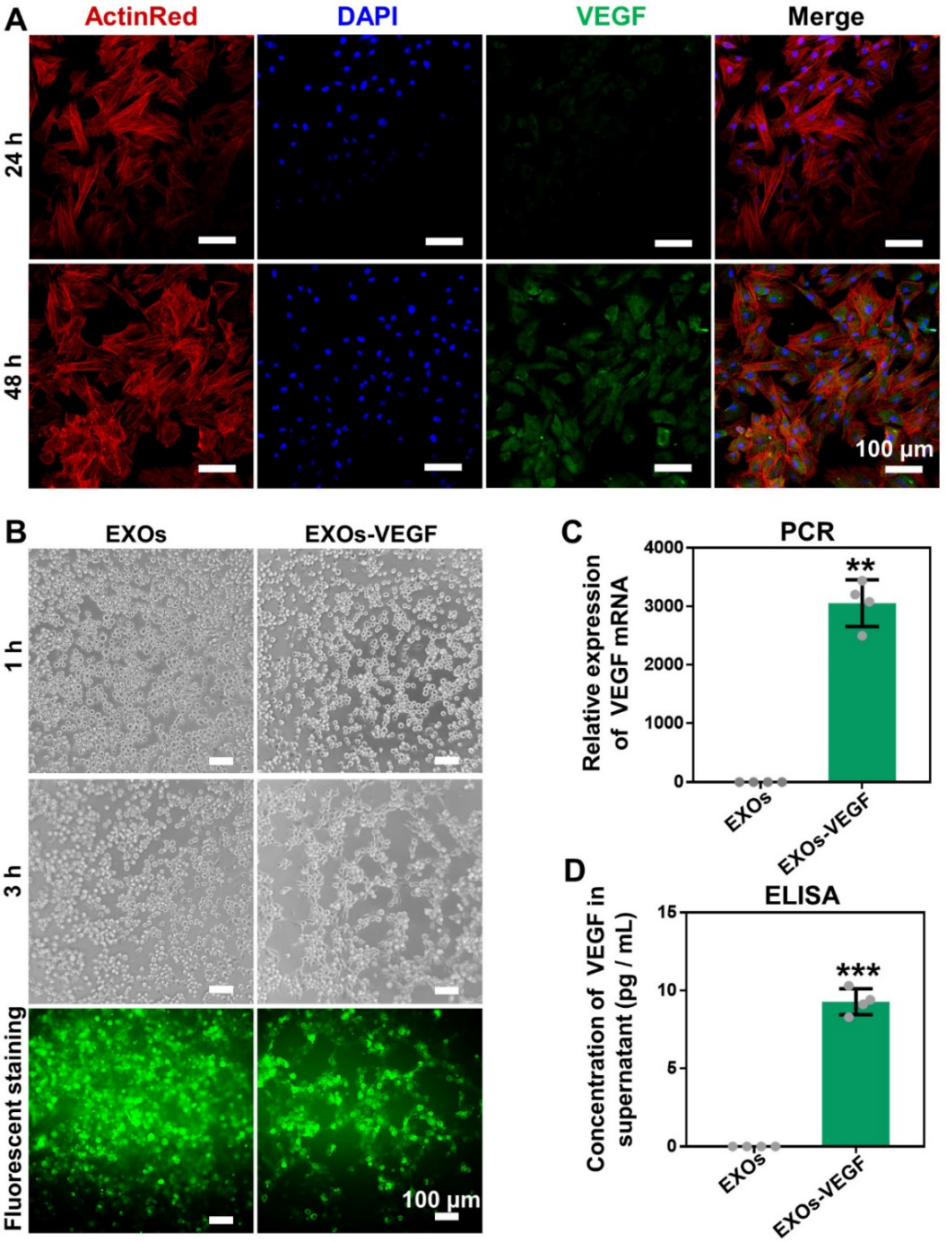
** Expression and angiogenetic effect of VEGF**. **(A)** The engineered exosomes (EXOs-VEGF) was successfully transfected into rBMSCs after culturing for 24 h and 48 h, and the positive gene expression of VEGF plasmid (pEGFP-kozVEGF165) was elevated with increased culture time. The nuclei of rBMSCs were stained with DAPI (blue), the cytoskeletons of rBMSCs were stained with ActinRed (red).** (B)** Tube formation assays with HUVECs was performed using the serum-free supernatant of rBMSCs with pure exosome (EXOs) and EXOs-VEGF for 1 h and 3 h. The results further demonstrated that the EXOs-VEGF extremely promoted tube formation of HUVECs *in vitro*. The cytoskeletons of HUVECs were stained with FITC (green).** (C, D)** Both qRT-PCR and ELISA clearly confirmed that the relative expression of VEGF in EXOs-VEGF was ultra-higher than that in pure EXOs (Independent-sample t-tests; **, *p* < 0.01; ***, *p* < 0.001 compared to EXOs group) (n = 4).

The plasmid gene of pEGFP-kozVEGF165 (VEGF) was verified by agarose gel electrophoresis; the main bands were located between 2000 bp and 3000 bp, which were consistent with the originally designed fragment **(Figure S1)**. The VEGF plasmid was subsequently encapsulated by exosomes via electroporation to generate the gene-activated engineered exosomes. After incubating the engineered exosomes with rBMSCs for 24 h and 48 h, we found that the transfection efficiency was significantly elevated with increased culture time** (Figure 3A** and **Figure S2)**. Tube formation assays with HUVECs further confirmed that the supernatants of the gene-activated engineered exosomes induced more tube formation than the pure exosome negative controls, and tube formation increased further when the culture time was increased from 1 h to 3 h **(Figure 3B** and **Figure S3)**. In addition, the relative expression of intracellular VEGF in the experimental group was approximately 2800-fold higher compared to the negative control based on qRT-PCR analysis** (Figure 3C)**. The concentration of secreted VEGF protein by enzyme linked immune sorbent assay (ELISA) was approximately 10 pg/mL, whereas secreted VEGF protein was undetectable in the control group** (Figure 3D)**.

### Characterization and modification of 3D-printed scaffolds

**Figure 4:**
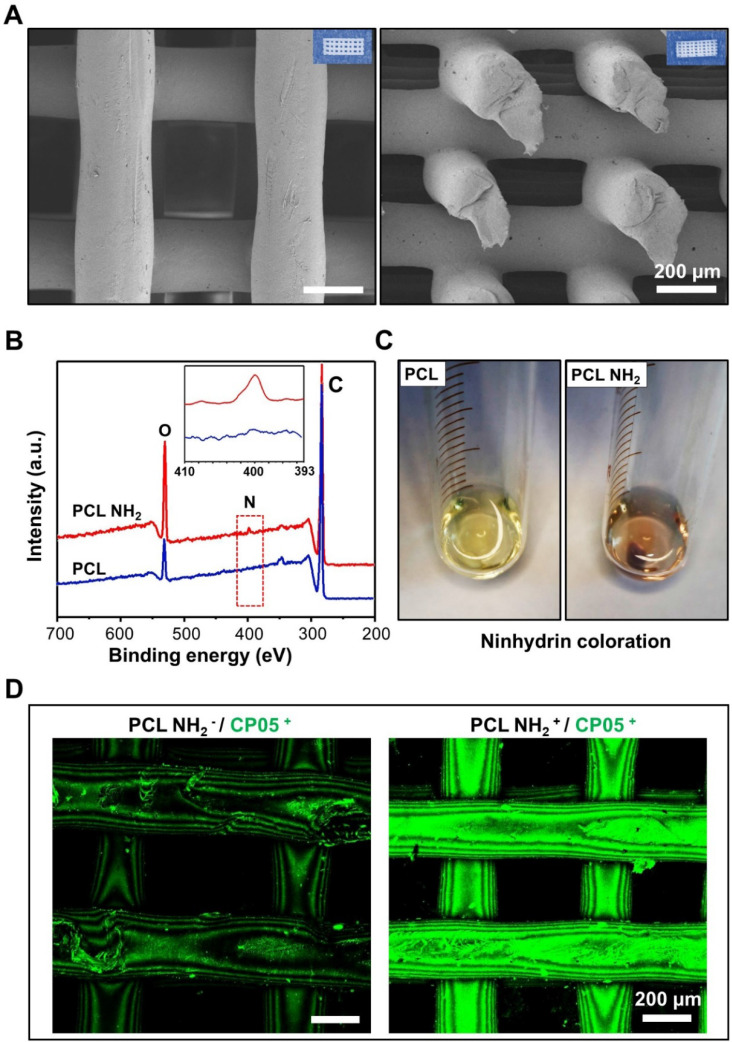
**Modification of 3D-printed PCL scaffolds. (A)** 3D-printed PCL scaffolds exhibited a highly intercommunicating porous morphology by SEM observation (Left image for the top view and Right image for the side view). **(B)** XPS analysis verified that the amino group (-NH_2_) was successfully coated onto the surface of 3D-printed PCL scaffold. **(C)** The amino groups between PCL and PCL-NH2 scaffolds were comparably analyzed by ninhydrin coloration. **(D)** The graft efficiency of the anchor peptide CP05 was obviously higher in PCL NH_2_^+^ than that in PCL NH_2_^-^. The CP05 was conjugated with Alexa Fluor 488 to present green fluorescence.

The 3D-printed scaffolds exhibited a micro-scale porous structure with pore diameters of approximately 250 μm. The structure was able to mimic that of trabecular bones, taking advantage of the exchange of nutrients and oxygen and the formation of neovascularization **(Figure 4A)**. The compressive strength of the 3D-printed PCL scaffolds was 4.8±0.6 MPa **(Figure S4)**. A positive charged amine group (-NH_2_) was first introduced to the 3D-printed scaffolds by 1,6-hexanediamine, and the XPS results clearly demonstrated a nitrogen peak detected for the 3D-printed PCL scaffolds modified with amine groups **(Figure 4B)**. In addition, the ninhydrin coloration assay indicated an obvious colour change before and after the amino group coating **(Figure 4C)**. All of these results verified that the amino groups were successfully coated onto the surface of the 3D-printed PCL scaffolds. To maintain a stable and flexible link between the engineered exosomes and the 3D-printed scaffolds, the CP05 exosomal anchor peptide used to modify the 3D-printed scaffolds. Our findings showed that the scaffolds modified with an amine group (PCL NH2^+^) exhibited a much greater absorption of CP05 than the scaffolds without the amine group modification (PCL NH2^-^). CP05 was conjugated to Alexa Fluor 488 for observation by confocal laser scanning microscopy **(Figure 4D)**. The graft efficiency of CP05 onto the PCL scaffolds was 26.7% (wt/wt).

### *In vitro* interaction between cells and scaffolds

**Figure 5:**
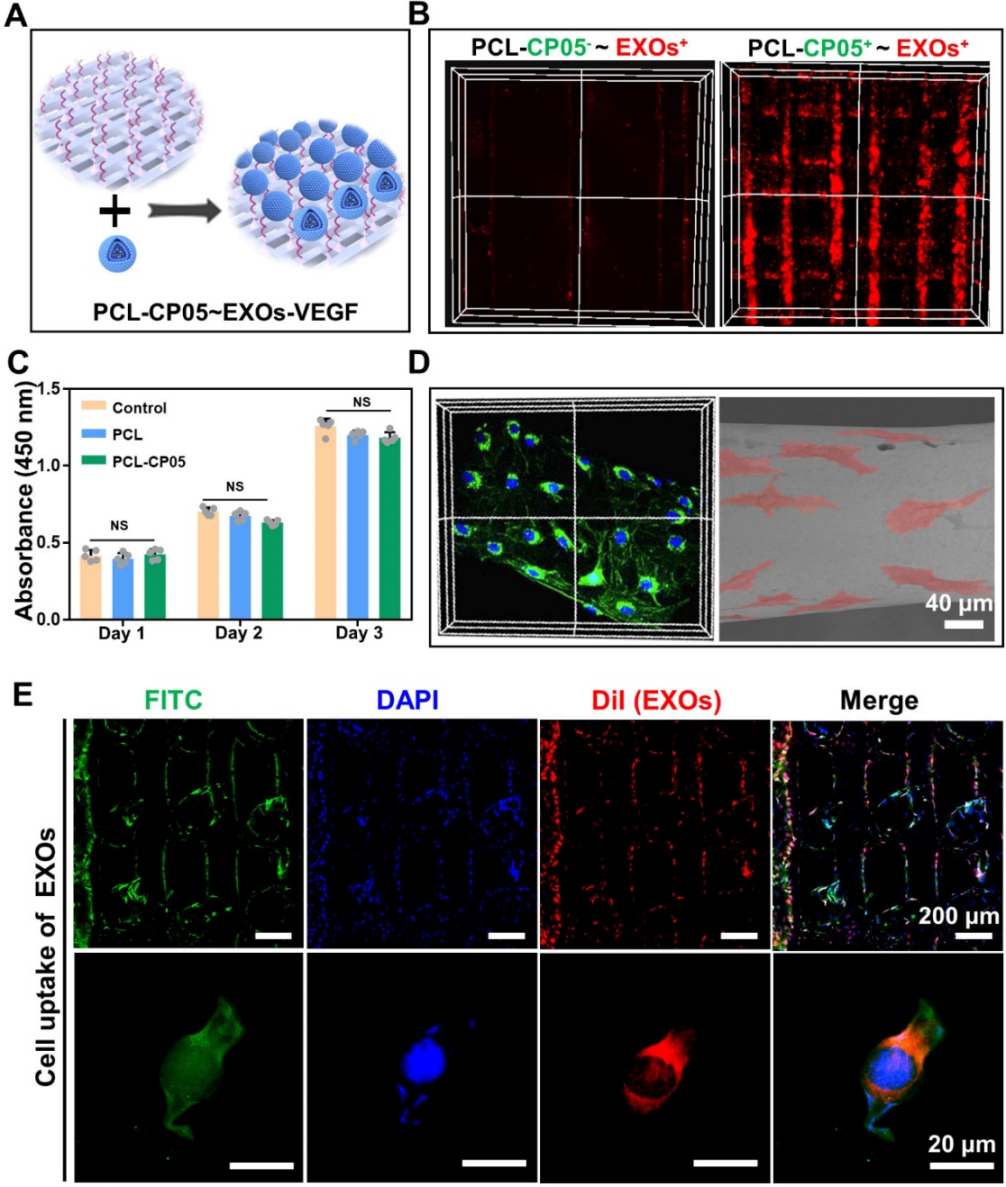
***In vitro* interaction between exosome-mediated bone scaffolds and rBMSCs. (A)** Schematic representation on the construction of the engineered exosome-mediated bone scaffolds via the linker of CP05 on combining nanoscale exosomes with macroscale bone scaffolds. **(B)** The graft efficiency of engineered exosomes connected onto 3D-printed bone scaffolds was significantly elevated with the help of the anchor peptide CP05. **(C)** Cell proliferation showed that there was no difference on biocompatibility before and after the modification with CP05. (one-way ANOVA followed by Tukey's post hoc test; NS, no significance compared to control group) (n = 4).** (D)** 3D-printed scaffolds were able to well support cell adhesion based on the analysis of confocal Z-stacks image and SEM image. **(E)** EXOs were internalized into 3D-printed scaffolds based on cell uptake. The nuclei of rBMSCs were stained with DAPI (blue), the cytoskeletons of rBMSCs were stained with FITC (green), and the EXOs were stained with DiI (red).

3D-printed PCL scaffolds modified with the CP05 anchor peptide (PCL-CP05) exhibited a higher affinity for the engineered exosomes than the control scaffolds without the CP05 modification **(Figure 5A-B)**. The CCK-8 assay results showed that there was no significant difference among the different groups at the same time point **(Figure 5C)**. In addition, the scaffold surface was able to support cell adhesion and spreading, indicating good biocompatibility of the 3D-printed PCL scaffolds **(Figure 5D)**. The graft efficiency of the engineered exosomes onto the PCL scaffolds was 41.7% (wt/wt). Cellular uptake assays showed that a large number of DiI-labelled exosomes were internalized and distributed in the perinuclear region of rBMSCs **(Figure 5E)**.

### Osteogenic differentiation induced by engineered exosomes

**Figure 6:**
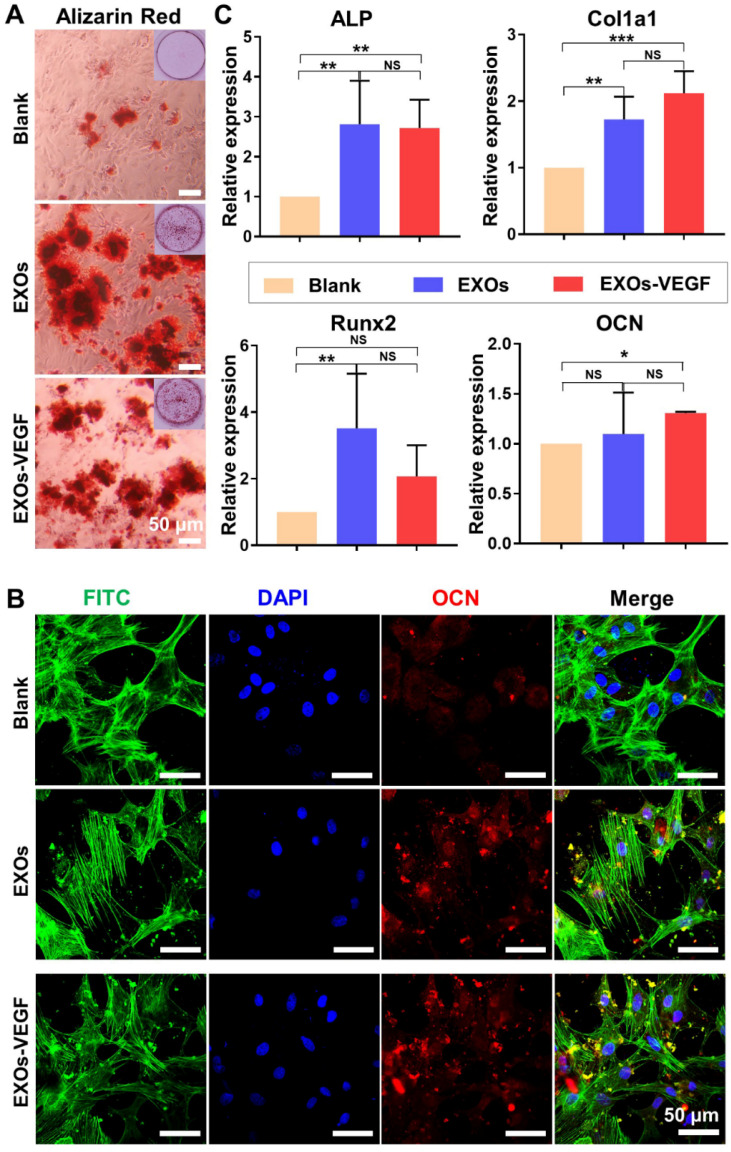
**ATDC5-derived exosomes promoted osteogenic differentiation of rBMSCs.** (**A**) Alizarin red staining showed EXOs could stimulate the formation of mineralization extracellular matrix of rBMSCs. (**B**) The osteogenic marker OCN was positively stained with Immunofluorescence staining, and cell nuclei and cytoskeletons were stained with DAPI (blue) and FITC-phalloidin (green), respectively. (**C**) QRT-PCR analysis of ALP, Col1a1, Runx2 and OCN further confirmed that both EXOs and EXOs-VEGF were able to promote osteogenic differentiation of rBMSCs and there was no significant difference between EXOs and EXOs-VEGF (one-way ANOVA followed by Tukey's post hoc test; *, *p* < 0.05; **, *p* < 0.01; ***, *p* < 0.001 compared to blank group) (n = 3).

ALP staining has been widely assessed as an early maker of osteogenic differentiation. Our findings showed there was no obvious difference in the ALP staining of each group on day 7 **(Figure S5)**. However, the alizarin red staining showed obvious mineralized nodules in both the exosomes and the engineered exosomes on the 14 day time point **(Figure 6A** and **Figure S6)**. There was also positive immunefluorescent staining for the osteogenic marker OCN on the 14 day time point **(Figure 6B** and **Figure S6)**. The qRT-PCR results further indicated that both the ATDC5-derived exosomes and the VEGF engineered exosomes could promote a certain degree of rBMSC osteogenic differentiation on day 7** (Figure 6C)**. Among the genes analysed, the expression levels of ALP and Col1a1 were significantly upregulated in the exosome-mediated cultures. There were no major differences in the expression of Runx2 and OCN between the ATDC5-derived exosomes and the engineered exosomes with encapsulated VEGF. All of these results implied that the ATDC5-derived exosomes enhanced the osteogenic capacity, however, the introduction of VEGF into the engineered exosomes did not obviously affect their osteogenic tendency.

### *In vivo* evaluation of osteogenesis and angiogenesis

**Figure 7:**
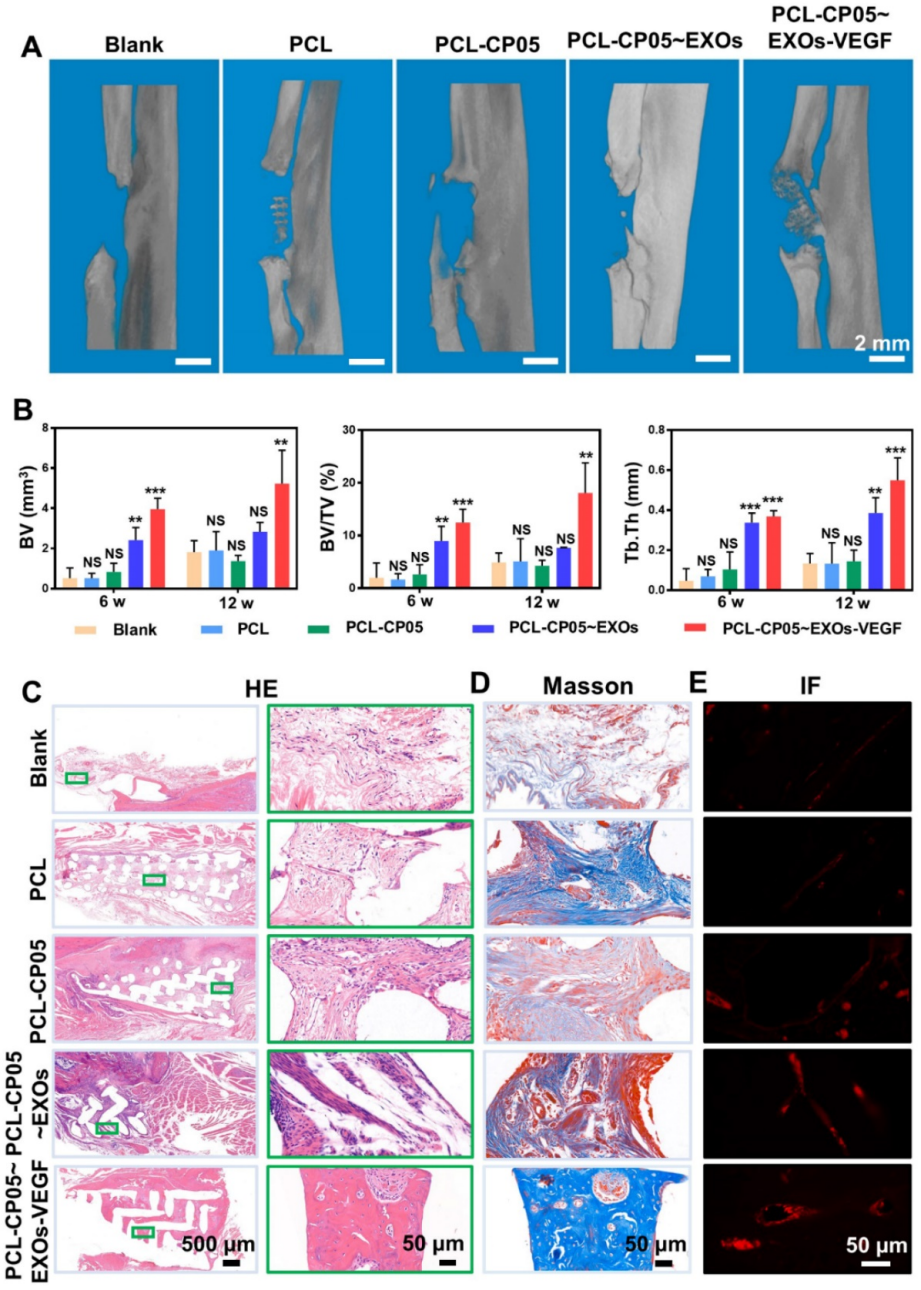
**Micro-CT and histological assessment of rat radial defect repair *in vivo*. (A)** 3D reconstructed micro-CT images showed the experimental group of PCL-CP05~EXOs-VEGF exhibited the best repair and induced to form a bulk of new bone at 12 weeks after implantation. **(B)** Quantitative comparison of BV, BV/TV and Tb.Th presented the consistent results (one-way ANOVA followed by Tukey's post hoc test; NS, no significance; *, *p* < 0.05; **, *p* < 0.01; ***, *p* < 0.001 compared to blank group) (n = 5). **(C)** HE staining and **(D)** Masson staining clearly showed the formation of new bone in the experimental group of PCL-CP05~EXOs-VEGF at the endpoint 12 weeks. Right panels presented the enlargement views of the green rectangles in the left panels. All of these results solidly demonstrated that the experimental group could significantly stimulate the new bone forming. **(E)** Immunofluorescence staining of the angiogenic marker CD31 exhibited a positive staining at 12 weeks after implantation.

We continued to investigate the performance of the gene-activated engineered exosomes *in vivo* using a rat radial defect model **(Figure S7A)**. Twelve weeks after implantation, the scaffold was integrated into the native bone tissue **(Figure S8A)** and many newly formed tissues filled in the scaffold pores **(Figure S8B)**. In addition, the compressive strength of implanted PCL scaffolds was greatly enhanced due to the newly formed bone at 12 weeks after implantation **(Figure S4).** Reconstructed micro-CT images revealed that the bone regeneration in the experimental group was significantly better than that in the other groups at the 6 and 12 week time points** (Figure 7A** and**Figure S7B)**. More importantly, compared with other groups, a bulk mass of new bone was generated only in the experimental group at 12 weeks after implantation. Additionally, the micro-CT images were quantified, including the bone volume (BV), bone tissue volume to total tissue volume ratio (BV/TV), and trabecular thickness (Tb.Th), and the results were highly consistent with the 3D reconstructed micro-CT images **(Figure 7B)**.

HE staining further indicated the presence of a bulk of newly formed bone tissue and a number of blood vessels were observed in the experimental group at the endpoint 12 weeks after scaffold implantation **(Figure 7C** and**Figure S7C)**. In contrast, the control group scaffolds were mainly filled with soft connective tissues consisting of fibrous connective tissue with randomly oriented low-density collagen fibres and blood vessels. Masson's Trichrome staining further indicated that more mature collagen fibres were present in the experimental group compared to the control groups **(Figure 7D** and **Figure S7D)**. The experimental group also had positive staining for the angiogenic marker CD31 by immunofluorescence **(Figure 7E** and **Figure S7E)**. All of these results solidly demonstrated that the well-designed, engineered, exosome-activated scaffolds were able to successfully induce vascularized osteogenesis.

## Discussion

Acellular therapy has attracted increasing attention due to its ability to bypass some of the inherent issues associated with conventional cell-based therapy, such as the cell source, cell bioactivity, cell immunity, long therapeutic times and high costs. Recent progress in cell-free therapies has highlighted the potential use of exosomes as a replacement for functional cells. Lin and colleagues explored the therapeutic effect of MSC-derived exosomes in a 3D-printed scaffold for early OA therapeutics, and demonstrated that MSC-derived exosomes could enhance mitochondrial biogenesis both *in vitro* and *in vivo* 31. Yang *et al.* found that a high fat diet altered the miRNA profile of visceral adipose tissue-derived exosomes to exacerbate colitis severity via the presence of proinflammatory miRNAs in high fat diet fed mice 32. Xu and his group found that the exosomes derived from clear cell renal cell carcinoma (CCRCC) patients transported miR-19b-3p into CCRCC cells and were able to initiate the EMT, promoting metastasis 33. Functional exosomes from the ATDC5 chondrogenic progenitor cell line have been verified to exhibit significant osteogenic differentiation capacity 18. In addition, ATDC5 as a mature cell line has been extensively used in bone tissue engineering due to its quick proliferation and stability in culture. Thus, here, we attempted to explore cell-free tissue engineering by constructing novel engineered exosomes that can be used as both an osteogenic matrix and a gene vector **(Figure 1)**. Our findings show that the ATDC5-derived exosomes exhibit osteogenic capacity similar to that of ATDC5 cells, and they can enhance the osteoblastic differentiation of rBMSCs *in vitro* **(Figure 6)**. In addition, to strictly control the quality and stability of the exosomes, we applied several approaches involving a uniform cell source, culture parameters and isolation parameters with ultracentrifugation-based techniques. Therefore, nanoscale exosomes from functional cells may be considered an alternative bioactive molecule for cell-free enhanced therapy.

Vascularized osteogenesis plays a pivotal role in promoting the regenerative repair of segmental bone defects, as the lack of vasculature can cause severe necrosis in large bone defects 34-36. As a crucial growth factor, VEGF has been extensively utilized to induce the vasculature reconstruction 19, 20; however, most of the current work has primarily utilized the VEGF protein, which can cause some problems due to its easy degradation, short half-life, systematic toxicity, and high cost 37. Thus, we suggest using the VEGF gene in place of the VEGF protein. Furthermore, exosomes have been explored as excellent biovectors to deliver diverse genes and drugs in sustained and enhanced therapies 38, 39. In this study, we constructed a novel, engineered exosome by encapsulating the VEGF gene in native, progenitor cell-derived exosomes. After transfection of the engineered exosomes, both PCR and ELISA assays showed there was the significant difference between pure exosomes and VEGF-containing exosomes. Our results verified that both native exosomes and engineered exosomes can promote the osteogenic differentiation of rBMSCs to similar levels, indicating that the introduction of VEGF did not impact the osteogenic differentiation capability of native exosomes **(Figure 6)**. Additionally, compared to the other groups, including the exosome group, the engineered exosomes exhibited the best osteogenesis in the rat radial defect model. This indicated that the engineered exosomes facilitate both angiogenesis and osteogenesis *in vivo*. Consequently, our gene-activated engineered exosomes have dual functions as an osteogenic matrix and a gene vector.

3D-printed porous bone scaffolds are beneficial to promote the ingrowth of new tissues, and they provide a 3D space for vasculature remodelling 1, 40-43. In this study, we considered it a vital precondition that nanoscale exosomes were combined with micro-scale porous scaffolds. We eventually utilized the CP05 exosomal anchor peptide as a linker molecule to establish a stable and flexible connection between the engineered exosomes and the 3D-printed porous scaffolds **(Figure 5A)**. It has been documented that CP05 is a CD63-specific exosomal anchor peptide, while CD63 as an exosomal marker and a tetraspanin enriched on the exosome surface 29. Thus, CP05 paves a new avenue for exosome engineering due to its direct and effective modification, cargo loading, and exosome capture. Our results confirm that the CP05 modification greatly improves the grafting efficacy between the engineered exosomes and the bone scaffolds **(Figure 5B)**.

Topical delivery and controllable release of functional exosomes at the defect site are the primary challenges for segmental large bone defects 44-47. First, local therapy of engineered exosomes can bypass several of the hurdles associated with traditional intravenous injection, including the lack of accumulation at the defect site and the risk of obstructing some blood-rich organs 24-26. Second, compared to intravenous administration, the local release of functional exosomes via directed transplantation can greatly improve the treatment efficiency 45. In our current work, we combined engineered exosomes with 3D-printed bone scaffolds to achieve local therapy via the directed transplantation of exosome-mediated bone scaffolds. *In vivo* animal evaluations clearly demonstrated that the well-designed scaffolds could successfully induce vascularized bone regeneration **(Figure 7** and**Figure S7)**.

## Conclusions

Acellular enhanced therapy is a promising strategy, and its clinical application could bypass a series of issues associated with conventional cell-based therapy, including immunological rejection, bioactive maintenance, long therapeutic times and high costs. In this study, we designed and constructed engineered exosomes using ATDC5-derived exosomes with an encapsulated VEGF gene plasmid, which exhibited dual functions in inducing rBMSC osteogenic differentiation and in modulating the controlled delivery of the VEGF gene. The engineered exosomes were combined with 3D-printed porous bone scaffolds via a specific linker (the CP05 anchor peptide) to effectively increase osteogenesis and angiogenesis in segmental bone defects. Hence, our current work provides an alternative use for functional exosomes in replacing seed cells and constructing cell-free tissue engineering with similar and equivalent therapeutic potential for vascularized bone remodelling.

## Materials and Methods

### Isolation and characterization of ATDC5-derived exosomes

The mouse chondrogenic progenitor cell line, ATDC5, was purchased from BNCC (Suzhou, China). The ATDC5-derived exosomes were isolated and characterized as described before 17. In brief, the serum-free medium of ATDC5 was centrifuged at 300×g for 15 min and 2000×g for 20 min at 4 °C to remove cellular debris. The supernatant was filtered through a 0.22 μm filter (Millipore, Merck, Germany) and ultracentrifuged at 100000×g in a 70Ti rotor for 3 h to collect exosomes (Ultracentrifuge, Beckman Coulter, L-80 XP). The protein concentration of the exosomes was quantified by BCA protein assay kit (Beyotime, China). The morphology of the exosomes was visualized by transmission electron microscopy (TEM, Hitachi, Japan). Nanoparticle tracking analysis (NTA, Particle-Metrix, GA) was performed to measure the nanoparticle size and size distribution. The zeta potential distribution of the exosomes was further investigated by DLS (Zetasizer Nano ZS90, Malcern, UK). The expression of TSG101 and CD63 on exosomes was detected by western blot. The specific binding efficiency between exosomes and the exosomal anchor peptide (Alexa Fluor 488 conjugated CP05, Sigma-Aldrich) was identified by flow cytometry (CytoFLEX, Beckman Coulter, USA).

### Preparation of VEGF plasmid gene and its electroporation into exosomes

The plasmid gene of pEGFP-kozVEGF165 (VEGF) was a gift from Professor Kun Ma lab (Dalian University of Technology, China). The VEGF plasmid DNA was isolated and identified as previously reported 28. In brief, the VEGF plasmid was propagated in *E. coli* DH5α cells. A single isolated colony of *E. coli* DH5α from a freshly streaked plate was picked to inoculate an appropriate volume of LB medium containing the appropriate antibiotic, and then incubated for overnight with vigorous shaking (~300 rpm, 37 °C; shaking incubator). The VEGF plasmid was isolated and purified using the Endo-Free Plasmid Mini Kit II (OMEGA, USA) as described in the kit manual. The DNA concentration was determined using a NanoDrop 2000 Ultramicro spectrophotometer (Thermo, USA). For electroporation, 30 µg of exosomes and 10 µg of VEGF were mixed in 400 μL of electroporation buffer (1.15 mM potassium phosphate pH 7.2, 25 mM potassium chloride, 21% Optiprep) and subsequently electroporated at 1000 V, 5 ms with one pulse condition using a Gene Pulser Xcell Electroporation System (BioRad, USA). After electroporation, the engineered exosomes were purified at 25,000×g for 1 h at 4 °C.

### Transfection and expression of VEGF

The rBMSCs were seeded and transfected with exosomes with encapsulated VEGF (10 μg/mL) when 80% confluency was reached. The gene transfection of VEGF was observed via the expression of enhanced green fluorescence protein (EGFP), which was linked to the VEGF plasmid. Cell culture supernatants without the serum were collected to evaluate *in vitro* in HUVEC tube formation assays as previously reported 17. In addition, qRT-PCR and ELISAs were performed to quantify the expression of VEGF as previously reported 17.

### Fabrication and modification of 3D-printed scaffold

A three-dimensional (3D) model of the scaffold was created using Solidworks 2018 software. Polycaprolactone (PCL) wires with a molecular weight of 50 kDa (Sigma, US) were printed at a printing temperature of 180 °C and a hot bed temperature of 25 °C using a 3D printer (Allcct, China). The PCL scaffolds were precisely cut into smaller pieces for further analysis. The surface and cutting surface of the 3D-printed PCL scaffold was observed using a field emission scanning electron microscope (GeminiSEM 300, ZEISS, Germany).

The 3D-printed PCL scaffolds were coated with amino groups by immersing them in 10 mL of 10% (w/v) 1,6-hexanediamine solution for 1 h at 37 °C. After that, the scaffolds were gently washed with ultrapure water and dried in a vacuum oven at 30 °C overnight. The obtained scaffolds were subsequently confirmed by X-ray photoelectron spectrum analysis (XPS; Kratos, UK). As a qualitative analysis of the amino groups, ninhydrin coloration assays were carried out by immersing the scaffolds in 2 mL of 2% (w/v) ninhydrin solution for 30 min at 50 °C.

To graft the CP05 exosomal anchor peptide onto the 3D-printed PCL scaffolds, the amino group coated scaffolds were first incubated in 4 mL of a CP05 peptide solution (0.75 mg/mL) under the activation of 1.8 mg of 1-(3-dimethylaminopropyl)-3-ethyl carbonamide hydrochloride (EDC) and 2.7 mg of n-hydroxysuccinimide (NHS) at 37 °C overnight. The combination of the scaffolds and CP05 conjugated to Alexa Fluor 488 was measured by confocal laser scanning microscopy (FV3000, Olympus, Japan). Finally, the CP05 modified scaffolds were incubated with exosomes with encapsulated VEGF plasmid DNA to form gene-activated engineered exosomes.

### Evaluation of the biocompatibility between cells and scaffolds *in vitro*

Cell proliferation and adhesion assays were carried out to investigate the biocompatibility of the 3D-printed PCL scaffolds. Briefly, rBMSCs were seeded onto different scaffold substrates at 2000 cells/well in 96-well plates for 24 h, 48 h and 72 h. After that, 10 μL of cell counting kit-8 (CCK-8, YEASEN, China) reagent was added to each well and incubated for 1 h at 37 °C. The absorbance at 450 nm was measured with an EnVision Multimode Plate Reader (PerkinElmer, Massachusetts, USA). In addition, the rBMSCs were seeded onto 3D-printed PCL scaffolds and cell adhesion was evaluated by SEM and confocal laser scanning microscopy.

### Cellular uptake and intracellular internalization of exosomes

To further explore the intracellular internalization of exosomes, rBMSCs were seeded onto PCL scaffolds conjugated with DiI-labelled exosomes and incubated for 48 h. After washing three times in PBS, the rBMSCs were fixed in 4% paraformaldehyde for 15 min and then washed again. Cell nuclei were stained with DAPI for 10 min and the cytoskeletons of rBMSCs were stained with FITC for 1 h at 37 °C. The cellular distributions of the exosomes were imaged using confocal laser scanning microscopy.

### Exosome-mediated osteogenic differentiation *in vitro*

To investigate the exosome-induced osteogenic differentiation of stem cells, rBMSCs from passage 2 were cultured with osteogenic medium containing 5% FBS, 0.1 μM dexamethasone, 10 mM β-glycerophosphate and 50 μg/mL ascorbic acid. Meanwhile, 10 μg/mL of exosomes and engineered exosomes loaded with VEGF were separately set as the experimental groups. The medium and exosomes were changed every 2 days. After culture for 7 days, alkaline phosphatase (ALP) staining (BCIP/NBT solution, Ameresco, USA) was performed following the kit protocol. Alizarin red staining (Solarbio, China) was carried out after culturing for 14 days. The alkaline phosphatase (ALP), collagen type 1 (Col1a1), osteocalcin (OCN), and runt-related transcription factor 2 (Runx2) gene expression levels were assessed by qRT-PCR and normalized to GAPDH; the gene primers used are listed in **Table S1.** One representative marker of OCN (Proteintech, 1:500) was investigated with the analysis of immunofluorescence staining.

### Construction of animal model

To track the performance of engineered exosome-mediated bone scaffolds *in vivo*, forty male (SD) rats with an average weight of 180 g were used to perform the radial defect model. Briefly, the rats were first anesthetized by the isoflurane inhalation anaesthesia (RWD, Shenzhen, China; 2.0-2.5% concentration), and then a segmental defect (~5 mm long) was created in the central radius of each animal model. The experimental groups, PCL, PCL-CP05, PCL-CP05~EXOs (10 μg) and PCL-CP05~EXOs-VEGF (10 μg) were then loaded into the defect site. A blank control was also created by loading nothing into the defect site. The experiments for each group were repeated five times and each group was harvested at 6 weeks and 12 weeks after surgery. Animal experiments were carried out in compliance with the protocol approved by the Institutional Animal Care and Use Committee of HUST.

### Micro-CT and histological analysis

Radial specimens were fixed and scanned using a micro-CT scanner (Skyscan1176, BRUKER). 3D reconstruction of the images was performed with multimodal 3D visualization software (Inveon Research Workplace, Siemens, Germany). Bone volume (BV), bone tissue volume to total tissue volume ratio (BV/TV), and trabecular thickness (Tb.Th) of the specimens were calculated. Furthermore, the specimens in each group were harvested for histological analysis. Haematoxylin & eosin (HE) staining and Masson's Trichrome staining were performed to analyse the newly formed bone tissue in the defect section. For angiogenic analysis, the CD31 vascular marker (Servicebio, 1:200) was evaluated by immunofluorescence staining.

### Statistical analysis

Quantitative data are presented as the means ± standard deviation (SD). Independent-sample t-tests were used to compare the means between two different groups. Comparisons between more than two groups were analysed by one-way ANOVA followed by Tukey's post hoc test. Statistical analyses were performed using SPSS software version 21.0 and *p* < 0.05 was considered to be statistically significant. All semi-quantifications were assessed using ImageJ software at a high resolution.

## Supplementary Material

Supplementary figures and tables.
