# Supplementary material for: Progenitor cell-derived exosomes endowed with VEGF plasmids enhance osteogenic induction and vascular remodeling in large segmental bone defects
Source: Theranostics. 2021 Jan 1;11(1):397–409. doi: 10.7150/thno.50741 (PMC7681080; doi:10.7150/thno.50741)
Supplement: Supplementary file 1 — Supplementary figures and tables. [file thnov11p0397s1.pdf]

Supporting Information

Progenitor cell-derived exosomes endowed with VEGF plasmids enhance osteogenic induction and vascular remodeling in large segmental bone defects

Yao Zha[1,2#], Yawu Li[1,2#], Tianyi Lin[1,2], Jia Chen[1,2], Shengmin Zhang[1,2*], and Jianglin Wang[1,2*]

Table S1. Primers of qRT-PCR

Gene	Organism	Primername	Sequence (5'-3')
VEGF165	human	Ho-VEGF165-F	GAGGGCAGAATCATCACGAAG
		Ho-VEGF165-R	TCCTATGTGCTGGCCTTGGTGA
ALP	Rat	Ra-ALP-F	AACGTGGCCAAGAACATCATCA
		Ra-ALP-R	TGTCCATCTCCAGCCGTGTC
Col1a1	Rat	Ra-Col1a1-F	CTGCCCAGAAGAATATGTATCACC
		Ra-Col1a1-R	GAAGCAAAGTTTCCTCCAAGACC
Runx2	Rat	Ra-Runx2-F	ATCCAGCCACCTTCACTTACACC
		Ra-Runx2-R	GGGACCATTGGGAACTGATAGG
OCN	Rat	Ra-OCN-F	CAGTAAGGTGGTGAATAGACTCCG
		Ra-OCN-R	GGTGCCATAGATGCGCTTG
GAPDH	Human/rat/mouse	G-GAPDH-F	*CCATGTTCGTCATGGGTGTGAACCA*
		G-GAPDH-R	*GCCAGTAGAGGCAGGGATGATGTTC*

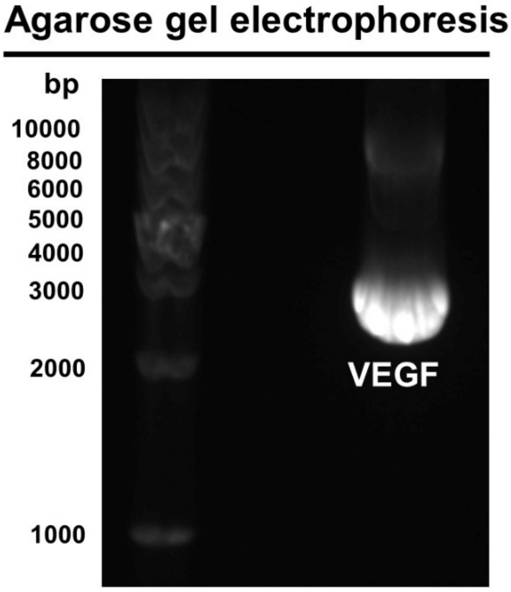

Figure S1. Identification of VEGF by agarose gel electrophoresis. The agarose gel electrophoresis showed that the main band of VEGF were located between 2000 bp and 3000 bp that were consistent with the inserted gene fragment.

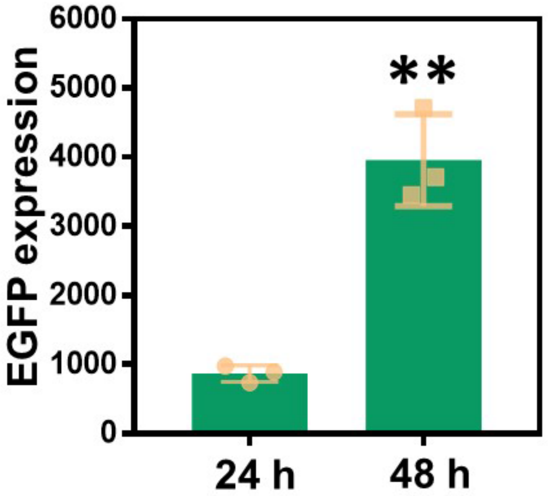

Figure S2. Semi-quantitative analysis of the fluorescence intensity of EGFP, as a reported gene, by ImageJ after transfected into rBMSCs for 24 h and 48 h. The results implied that the expression of VEGF plasmids was extremely improved with increased culture time (Independent-sample t-tests; **, $p < 0.01$ compared to 24 h group) ($n = 3$).

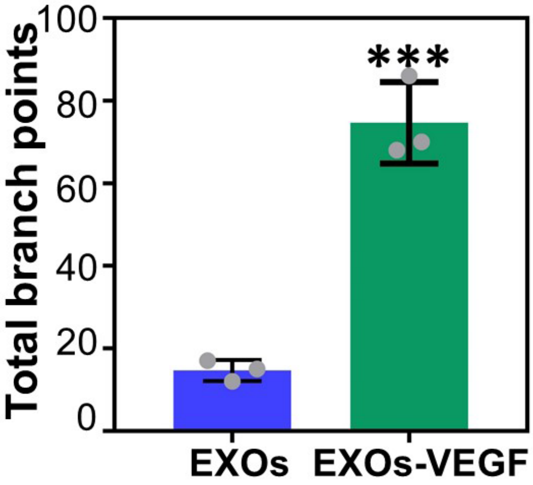

Figure S3. Semi-quantitative analysis of total branch points. There was a significant difference between EXOs and EXOs-VEGF (Independent-sample t-tests; ***, $p < 0.001$ compared to EXOs group) ($n = 3$).

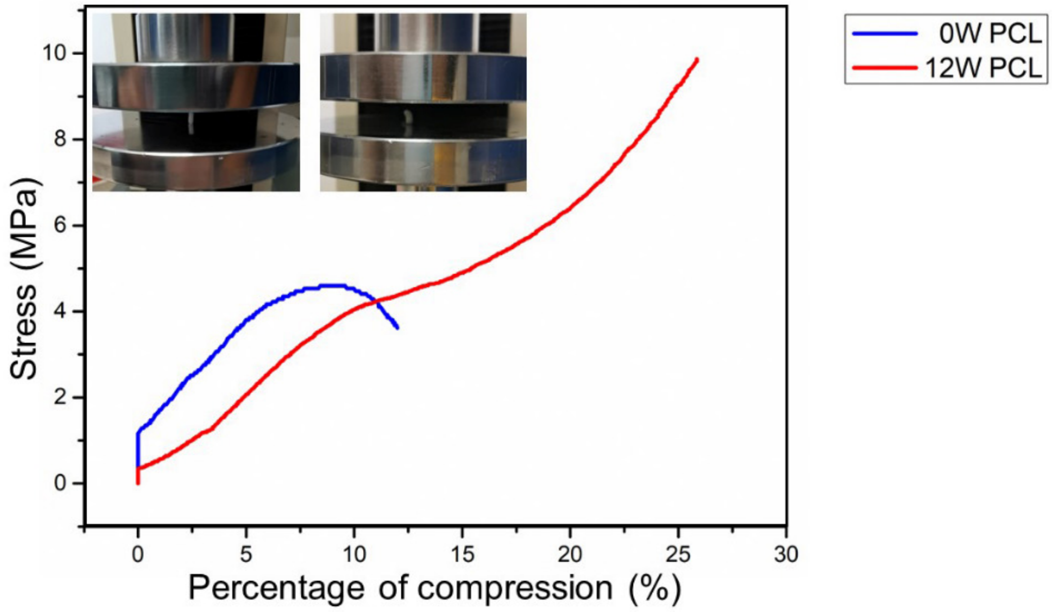

Figure S4. The compressive strength of pure 3D-printed PCL scaffolds (0W PCL) and PCL scaffolds after 12 weeks implantation in the radial defect model (12W PCL).

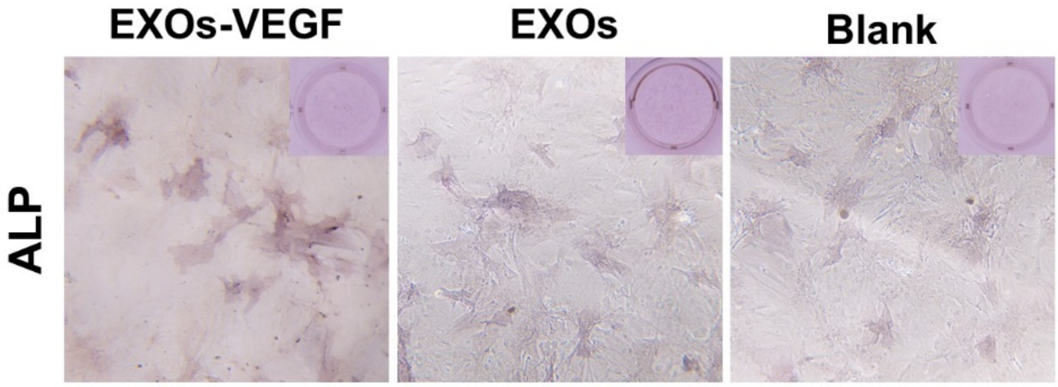

Figure S5. ALP staining showed that all groups could induce osteogenic differentiation at day 7. The experimental group of EXOs-VEGF exhibited a little bit deep staining compared with EXOs and the blank control. The blank control was treated with osteogenic medium.

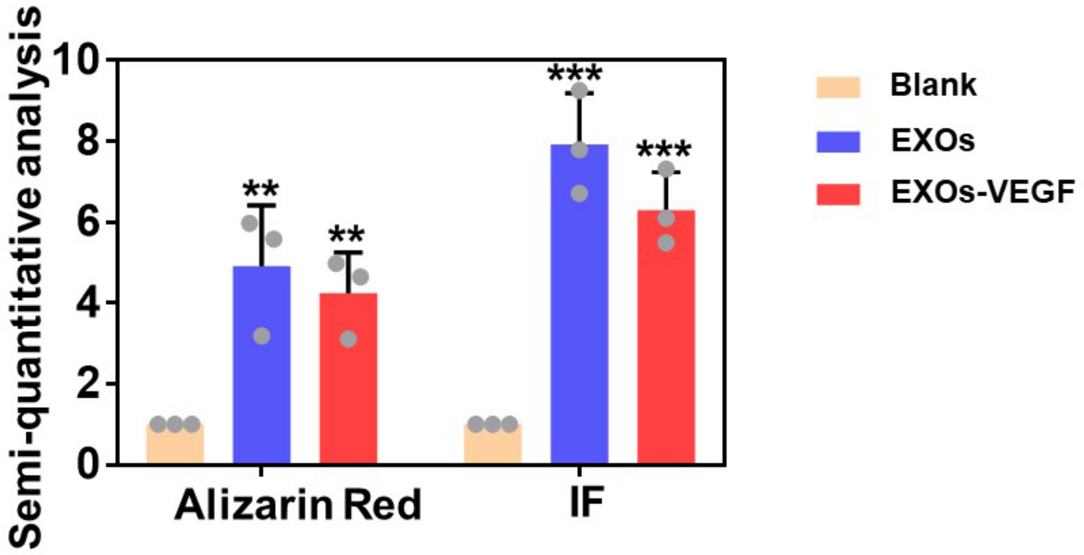

Figure S6. Semi-quantitative analysis of the Alizarin red staining and OCN immunofluorescence by ImageJ. There was no significant difference between EXOs and EXOs-VEGF (Independent-sample t-tests; **, $p < 0.01$; ***, $p < 0.001$ compared to blank group) ($n = 3$).

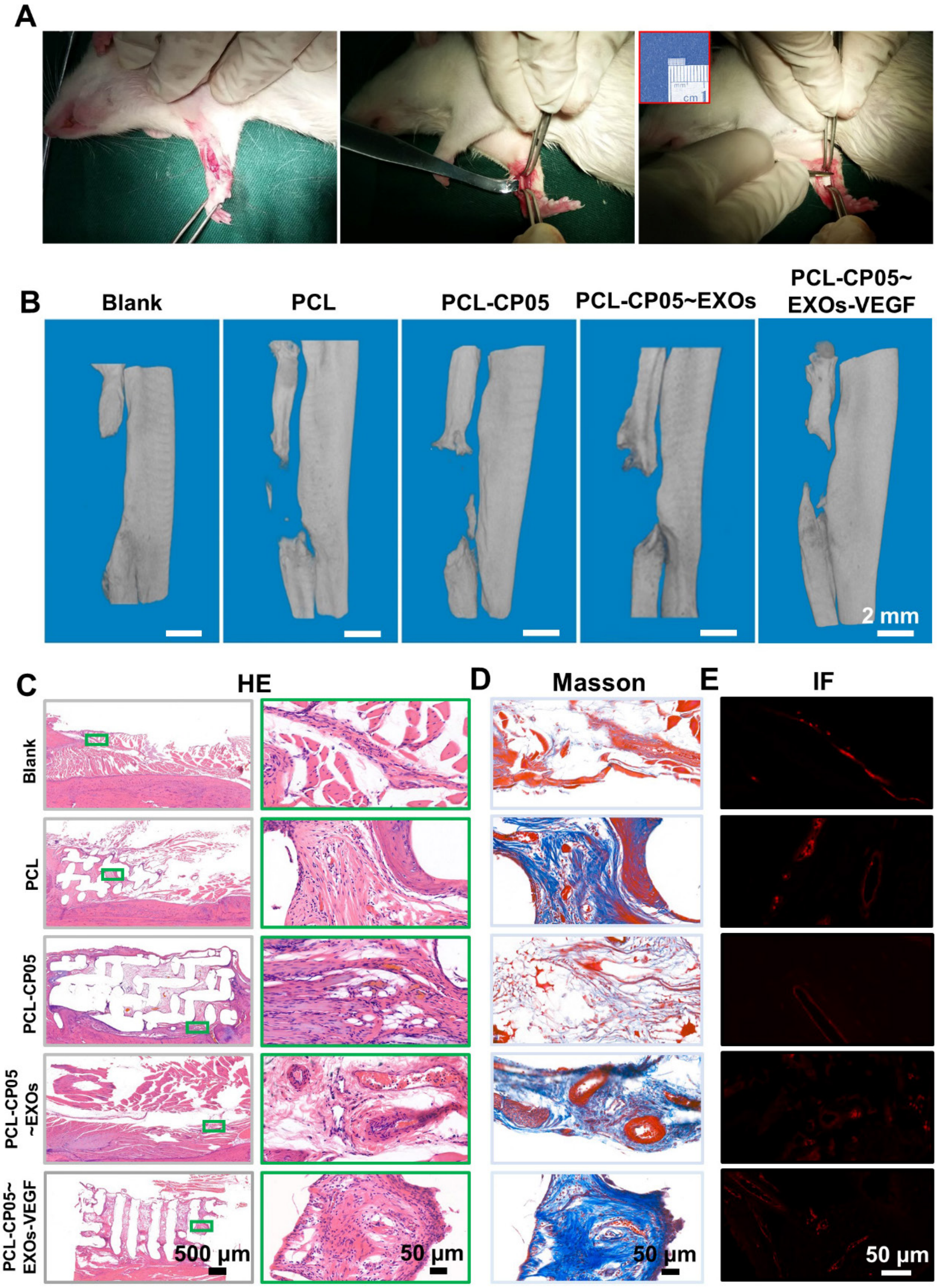

Figure S7. (A) The surgery drawing on the construction of rat radial defect model and scaffolds implantation. **(B)** 3D reconstructed micro-CT images of new bone at 6 weeks. Both **(C)** HE and **(D)** Masson staining demonstrated the formation of new bone in each group at 6 weeks after scaffold implantation. Right panels presented the enlarged views of the green rectangles in left panels. **(E)** Immunofluorescence staining of the angiogenic marker CD31 exhibited a positive staining after implantation for 6 weeks.

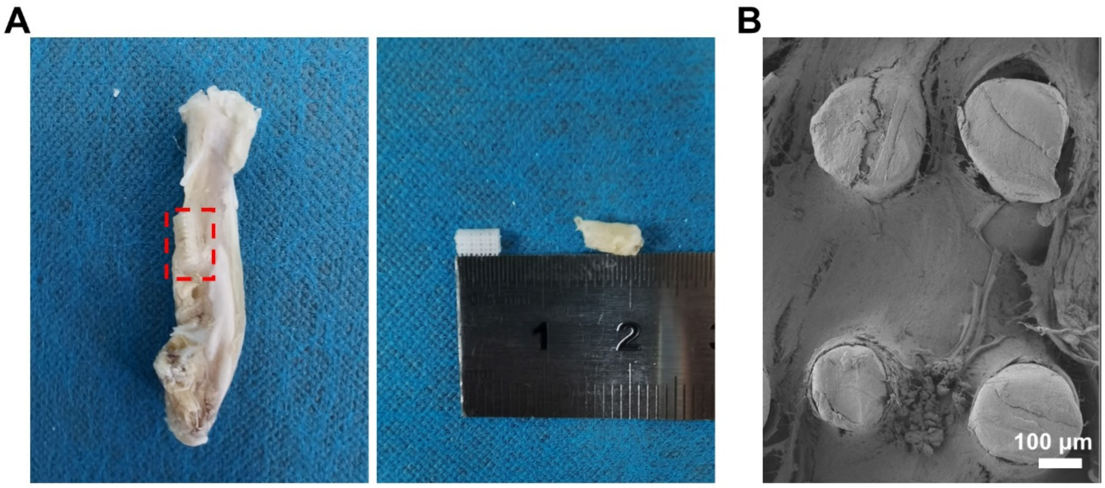

Figure S8. (A) The morphology of scaffold at 12 weeks after implantation in radial defect model. **(B)** SEM image of scaffold showed the porous scaffolds were filled with a bulk of fibrous connective tissue.
